# Application of *Nostoc sphaericum* and *Opuntia ficus-indica* Mucilage in the Coagulation–Flocculation Process of Sanitary Landfill Leachate: An Optimization Study

**DOI:** 10.3390/polym18040474

**Published:** 2026-02-13

**Authors:** Yudith Choque-Quispe, Aydeé M. Solano-Reynoso, Carlos Eduardo Dueñas-Valcarcel, Edwar Arostegui-Leon, Liliana Rodriguez-Cardenas, David Choque-Quispe

**Affiliations:** 1 Environmental Engineering Department, Universidad Nacional José María Arguedas, Andahuaylas 03701, Peru; 2 Research Group for the Development of Advanced Materials for Water and Food Treatment, Universidad Nacional José María Arguedas, Andahuaylas 03701, Peru; 3 Basic Sciences Department, Universidad Nacional José María Arguedas, Andahuaylas 03701, Peru; 4 Advanced Materials Research Laboratory for Water and Food Treatment, Universidad Nacional José María Arguedas, Andahuaylas 03701, Peru; 5 Food Nanotechnology Research Laboratory, Universidad Nacional José María Arguedas, Andahuaylas 03701, Peru; 6 Agroindustrial Engineering Department, Universidad Nacional José María Arguedas, Andahuaylas 03701, Peru

**Keywords:** biocoagulants, *Nostoc sphaericum*, *Opuntia ficus-indica*, Box–Behnken sanitary landfills, leachate

## Abstract

Leachates generated in sanitary landfills are a mixture of contaminants harmful to adjacent ecosystems. Coagulation and flocculation are common treatment methods; however, their efficiency depends on the type of coagulant–flocculant and the operating conditions. This study addressed leachate treatment using two natural biocoagulants, Nostoc sphaericum (CNS) and Opuntia ficus-indica mucilage (CMN), in combination with aluminum sulfate (CSA). Optimization was performed using response surface methodology, employing a Box–Behnken design with five factors, namely CNS, CMN, and CSA doses, as well as agitation time and agitation speed, evaluated at three levels, on turbidity reduction. Fourier transform infrared spectroscopy (FTIR) showed that the biocoagulants possess anionic surfaces with carboxyl and hydroxyl groups. The particle size of CNS exhibited a bimodal distribution with a zeta potential of −28.74 mV, while CMN showed a unimodal distribution with a zeta potential of −21.95 mV. Under optimal conditions (88.97 mg/L CNS, 105.60 mg/L CMN, 9.13 mg/L CSA, a mixing time of 25.96 min, and an agitation speed of 24.21 rpm), a turbidity reduction to 48.15 NTU was predicted. During the experimental validation of these optimal conditions, turbidity was reduced to 49.02 NTU, achieving a removal efficiency of 70%. Total organic carbon (TOC) was reduced by 65%, and metals such as arsenic were reduced by 56%. Reductions in phosphates and Biochemical Oxygen Demand (BOD_5_) were moderate, while the removal of Chemical Oxygen Demand (COD), surfactants, and ammoniacal nitrogen was limited. These results indicate that the combination of CNS and CMN is a viable alternative for leachate pretreatment.

## 1. Introduction

Changes in economic activities and people’s lifestyles are increasing global waste production [1,2]. It is estimated that by 2050, municipal solid waste generation will exceed 3.4 billion tons, representing a 70% increase over current levels [3]. The predominant practice of disposing of waste in landfills generates leachate, a complex and high-risk effluent. The composition of leachate varies depending on the age of the landfill and the type of waste, and it contains high concentrations of recalcitrant organic matter, such as humic and fulvic acids, heavy metals, salts, and organic compounds. This toxic mixture makes it a serious threat to aquifers, surface water bodies, public health, and surrounding ecosystems [4,5,6]. To mitigate this threat, various treatment technologies have been implemented. Leachate recirculation, while accelerating landfill stabilization, leads to operational problems such as odor emissions and high maintenance costs [7]. Biological treatments, while efficient for biodegradable organic matter, have significant limitations when treating recalcitrant pollutants and fluctuations in pollutant load. Similarly, advanced physicochemical processes, such as oxidation or nanofiltration, are effective, but their application is restricted by high energy requirements and operating costs [5,8,9,10].

Although coagulation and flocculation are often used as leachate pretreatment, they are generally preceded by equalization and pH adjustment to stabilize the effluent [11,12]. Their main function is to remove colloids and certain contaminants such as color, turbidity, and some metals, thus conditioning the effluent for subsequent processes. This improves the efficiency of biological treatments such as activated sludge, SBR, or MBBR, as well as advanced treatments like membrane filtration and reverse osmosis, which are highly susceptible to fouling and inhibition by recalcitrant contaminants [13,14,15].

However, the use of conventional chemical coagulants, such as aluminum or iron salts, generates large volumes of toxic sludge, which becomes a new hazardous waste that is difficult and costly to dispose of [1,2,10,15,16].

In addition, the use of metallic coagulants, such as aluminum, iron, and titanium salts, may cause pH alterations and the release of residual metals, potentially leading to secondary environmental impacts and increased operational costs. However, although recent studies have reported that titanium-based coagulants are efficient in contaminant removal, their higher cost and the difficulties associated with large-scale implementation limit their application in leachate treatment [17,18].

Sustainable solutions have been tested, and natural biopolymers have emerged as a promising alternative. Opuntia ficus-indica mucilage and Nostoc sphaericum cyanobacteria have proven to be particularly effective [5,19,20]. The mechanism of action is based on a combination of charge neutralization, adsorption, and bridging, facilitated by the polysaccharides and functional groups of the biocoagulants. Polysaccharides contain carboxyl and hydroxyl groups, which interact with contaminants in the leachate by neutralizing the negative charges of suspended particles, allowing them to agglomerate [21,22,23]. At the same time, the polymer chains act as bridging agents, trapping and aggregating the already destabilized particles to form larger, heavier flocs that settle easily. A sweeping mechanism may also occur, where the biopolymer forms a network that drags and removes contaminants as they precipitate [24,25,26,27]. However, despite its potential, optimizing the dosage and operating conditions for mixing these biopolymers with traditional coagulants in leachates remains an under-researched area. This study proposes a combination of natural biopolymers, namely Nostoc sphaericum (CNS) and Opuntia ficus-indica (CMN), with aluminum sulfate (CSA) in the coagulation and flocculation process. To this end, a Box–Behnken design (BBD) was used, a robust statistical tool within response surface methodology (RSM) that allows the interactions between coagulant dose, time, and agitation speed to be evaluated with a reduced number of experiments [1,5,10,28,29,30,31]. The objective of the study was to achieve the highest turbidity removal under optimal conditions, but at the same time to validate these optimal conditions with the removal of organic matter, nutrients, heavy metals, and surfactants, in order to develop a viable alternative to conventional treatment systems, contributing to sustainable waste management and environmental protection.

## 2. Materials and Methods

### 2.1. Leachate Sampling and Characterization

The leachate was collected from the Andahuaylas sanitary landfill, located on San José Hill, Apurímac Region, Peru (13°39′50.67″ S, 73°21′50.50″ W). The landfill operates with geomembrane-lined cells and anaerobic ponds for leachate collection and receives approximately 27.4 t/day of solid waste [26]. Composite leachate samples were collected from the main collector of the sanitary landfill on six occasions between April and June 2025, following the methodology described in [27].

The physical and chemical characterization of the leachate was performed according to the methods listed in Table 1. For heavy metal analysis, samples were filtered through a 0.45 µm membrane and analyzed in triplicate using an inductively coupled plasma optical emission spectrometer (ICPE-9820, SHIMADZU, Tokyo, Japan). Analyses were conducted in an axial mode with a plasma exposure time of 30 s, a gas flow rate of 10 L/min, and a rinse speed of 60 rpm between samples [26].

### 2.2. Raw Materials and Reagents

Cladodes of Opuntia ficus-indica were collected in the district of Santa Rosa Talavera, Andahuaylas Province, Peru, located at 13°39′01″ S, 73°26′29″ W, at an altitude of 2800 m above sea level, during the period from September to November 2024. The samples were transported in sterile containers and stored under controlled conditions to preserve their properties. The cyanobacterium Nostoc sphaericum was collected from the Huamanilla high Andean lagoon, located at 13°47′52″ S, 73°17′55″ W, at an altitude of 4251 m above sea level, in Andahuaylas Province, Peru, during the period from March to April 2024. Analytical-grade aluminum sulfate (CSA) was also used (Sigma-Aldrich, St. Louis, MO, USA).

### 2.3. Obtaining Biocoagulants

**Figure 1:**
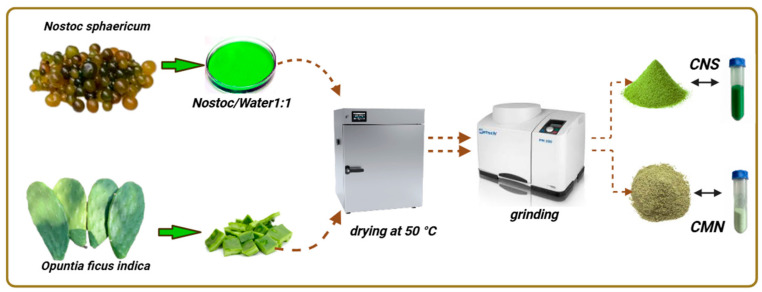
Schematic of the extraction process for the CNS and CMN biocoagulants.

Cladodes of *Opuntia ficus-indica* were washed, peeled, and cut into cubes. The material was then dried at 50 °C for 15 h [23] and finely ground using a planetary ball mill (QXQM series) at 50 rpm, obtaining the biocoagulant CMN (Figure 1).

Similarly, *Nostoc sphaericum* was washed with distilled water and mechanically homogenized in a blender with distilled water at a 1/1 *p*/*v* [34]. The extract was sieved through a 63 µm mesh; the filtrate was then dried in silicone molds at 50 °C for 15 h, and the dried material was ground at 50 rpm in a planetary mill (QXQM series), obtaining the biocoagulant CNS (Figure 1).

In both cases, the extraction was based on previous studies indicating that the preservation of naturally soluble polysaccharides and functional groups can be achieved through drying and mechanical milling, and that acid- and base-free methods represent a more sustainable alternative [35,36,37].

### 2.4. Methods for Characterization of the Biocoagulants

#### 2.4.1. FTIR Analysis

Fourier transform infrared (FTIR) spectroscopy was used to identify the functional groups present in the biocoagulants. The analyses were carried out using a Thermo Fisher spectrometer (Nicolet IS50 model) (Thermo Fisher Scientific, Waltham, MA, USA). Powdered samples were mixed with IR-grade KBr to prepare pellets and analyzed in transmission mode over the range of 4000–400 cm^−1^ with a resolution of 4 cm^−1^ [33,34].

#### 2.4.2. Determination of Particle Size and Zeta Potential

To determine particle size and zeta potential (ζ), 4 mg of each biopolymer sample were dispersed in 5 mL of ultrapure water and stirred at 1000 rpm for 5 min. The suspension was then sonicated for 10 min to achieve a homogeneous dispersion. The hydrodynamic diameter and particle size distribution were measured by dynamic light scattering (DLS) using a Nicomp Nano ZLS Z3000 instrument (Entegris, Billerica, MA, USA) at a scattering angle of 90°. Zeta potential (ζ) was determined using the same instrument by analyzing 2 mL of the dispersion at a wavelength of 632.8 nm and a scattering angle of −14.14 [27,34].

#### 2.4.3. Determination of the Point of Zero Charge

To determine the point of zero charge (PZC), 50 mL solutions containing 0.05 g of each biopolymer were prepared. The initial pH was adjusted over a range from 2 to 12 using 0.1 N NaOH and HCl solutions. The suspensions were stirred at 150 rpm for 24 h, after which the final pH was measured. The PZC was determined as the intersection point obtained by plotting the final pH versus the initial pH [34].

### 2.5. Experimental Procedure

#### Experimental Design and Process Configuration

The coagulation tests were conducted following a Box–Behnken experimental design, which allowed for the evaluation of the combined effects of five independent variables at three levels (Table 2) [1,38,39,40].

The selection of independent variables and their experimental ranges was based on preliminary jar test trials and operational criteria commonly used in coagulation–flocculation processes. The doses of CNS, CMN, and aluminum sulfate, as well as the mixing times and stirring speeds, were defined to ensure effective destabilization and adequate floc formation, avoiding overdosing. Similar ranges are applied in the treatment of leachates and complex wastewater [10,16].

The experimental design included 46 experiments and was carried out using a Jar-Test instrument (PB-900; Phipps & Bird, Richmond, VA, USA). 500 mL of untreated leachate was added to each jar. During the first rapid mixing stage, which lasted 1 min at 120 rpm, the biocoagulants and coagulants (CNS, CMN, and CSA) were added in doses corresponding to those defined in the experimental design.

After the rapid mixing stage, the agitation speed was reduced, and the mixing time and agitation speed were adjusted according to the levels specified in Table 2, in order to promote particle aggregation. Subsequently, the suspensions were allowed to settle for 1 h. At the end of the settling period, samples were collected from the supernatant, and turbidity, pH, and electrical conductivity were measured. These parameters were selected as response variables for process optimization.

For the generation and analysis of the empirical models, according to Equation (1), turbidity, pH, and electrical conductivity (EC) of the treated leachate were considered response variables.

(1) $$Y=\beta_{0}\sum_{i=0}^{k}\beta_{i}X_{i}+\sum_{i=1}^{k}\beta_{ii}X_{i}^{2}+\sum_{i>j}^{k}\cdot \sum_{j}^{k}\beta_{ij}X_{i}X_{J}+e$$

where *Y* is the predicted response; *β*_0_, *β_i_*, and *β_ii_* are the linear, quadratic, and interaction coefficients, respectively; *X_i_* and *X_j_* are the independent variables; *e_i_* is the residual error; and *k* is the number of variables.

The models were validated using analysis of variance (ANOVA) with a significance level of *p* < 0.05. The model was considered adequate when the lack-of-fit test was not significant (*p* > 0.05).

To evaluate the goodness of fit and predictive capability, the coefficient of determination (R^2^), adjusted R^2^, predicted R^2^, and adequate precision were employed. R^2^ was used to assess the agreement between the model and the experimental data; the adjusted R^2^ accounted for the number of predictors in the model; the predicted R^2^ was used to estimate the predictive performance; and adequate precision was used to evaluate the model’s ability to distinguish the real effects of the factors.

The model was considered valid when it presented an R^2^ value close to 1, an adjusted R^2^ consistent with the R^2^, an absolute difference ≤ 0.2 between the adjusted R^2^ and the predicted R^2^, and adequate precision values greater than 4 [41,42,43]. Statistical analyses were performed using Design-Expert software (v. 7.0.0, demo version).

### 2.6. Optimization and Experimental Validation

Process optimization was performed using the desirability function in order to identify the operating conditions that minimize turbidity while maintaining the pH within the range of 6.0–9.0 and ensuring that the EC was equal to or lower than the initial value of the leachate.

To validate the predictive capability of the model under the identified optimal conditions, experimental coagulation–flocculation tests were conducted. Treatment performance was evaluated by measuring the responses (turbidity, pH, and EC), and to determine efficiency of the treatment under these optimal conditions, the percentage of removal of BOD_5_, COD, TOC, metals, ammoniacal nitrogen, phosphates, and surfactants was quantified. The removal efficiency for each parameter was calculated according to Equation (2).

(2) $$\%R=\frac{T_{0\;}{-\;T}_{f}}{T_{0}}\times 100$$

where T_0_ is the initial concentration, and T_f_ is the final concentration.

### 2.7. Statistical Techniques

The results are reported using the arithmetic mean and standard deviation of the data obtained in triplicate. To evaluate significant differences between treatments, Student’s *t*-test for paired data was applied with a significance level of 5%. The data were analyzed using Statistica software, version 12.

During the preparation of this manuscript, the authors used generative artificial intelligence tools to support the translation process. The manuscript was reviewed and finalized by the authors, who assume full responsibility for its content.

## 3. Results and Discussion

### 3.1. Characterization of the Initial Leachate

The initial leachate presented a pH of 8.01, which is within the range of 6 to 9, characteristic of leachates in an intermediate stage that are in a transition phase between young and mature leachates (Table 3) [44,45]. The COD reached 817 mg/L, while the BOD_5_ was 563 mg/L, exceeding the permissible limits of 400 mg/L and 20 mg/L, respectively [44]. The BOD_5_/COD ratio of 0.69 indicates a highly biodegradable organic fraction, which makes biological treatment possible.

In addition, a high NH_3_-N concentration of 640 mg/L, exceeding the limit of 5 mg/L, and a turbidity of 164.8 NTU indicate that the leachate is complex and less stable. Furthermore, heavy metals such as Cd (0.48 mg/L), As (0.75 mg/L), and Pb (0.38 mg/L) exceeded the permitted limits [44].

Although the biodegradability index suggests the feasibility of biological treatment, the raw leachate presents high metal concentrations, indicating the need for physicochemical treatment to reduce the contaminant load. Despite the relatively high BOD_5_/COD ratio, the coagulation–flocculation process is therefore proposed as a pretreatment step to reduce turbidity, colloidal organic matter, and inorganic components that could affect the performance of subsequent biological treatment.

### 3.2. Characterization of the Biocoagulants

#### 3.2.1. FTIR Analysis of Biocoagulants

**Figure 2:**
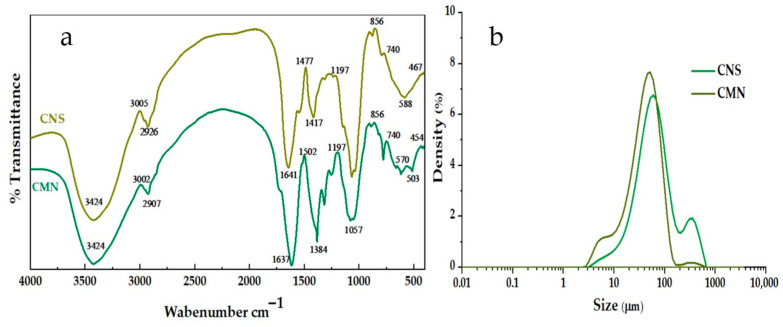
(**a**) FTIR spectra and (**b**) particle size distribution of CNS and CMN samples.

FTIR spectroscopy was used to analyze the presence of active functional groups in CNS and CMN (Figure 2a). The broad band around 3424 cm^−1^ is associated with O-H stretching of hydroxyl groups, which are characteristic of polysaccharides and participate in the formation of hydrogen bonds with water molecules and contaminants, favoring particle binding and adsorption processes [45]. The bands observed at 2926 cm^−1^ for CNS and 2907 cm^−1^ for CMN correspond to C-H stretching of methyl and methylene groups, indicating the presence of a polymeric chain of an aliphatic nature [46,47].

The bands located at 1637 and 1641 cm^−1^ are associated with carboxyl groups (-COOH), which, upon dissociation in water, confer an anionic character to the surface of the biocoagulants [48]. This negatively charged surface favors electrostatic interactions with positively charged species, such as metallic cations, and, together with the presence of hydroxyl groups, promotes particle agglomeration through the formation of hydrogen bonds [49,50]. Moreover, the bands at 1384 cm^−1^, 1477 cm^−1^, and 1057 cm^−1^ also indicate the presence of C-O and C-OH groups, characteristic of polysaccharides. The presence of these functional groups suggests their potential involvement in the coagulation–flocculation process, as they may contribute to contaminant removal through electrostatic interactions, hydrogen bonding, and polymer bridging mechanisms, as reported in previous studies [21,27,34,45,51,52].

#### 3.2.2. Particle Size Analysis of the Biocoagulants

The efficacy of natural coagulants depends directly on particle size. CNS exhibits a bimodal particle size distribution of approximately 5 µm and 100 µm (Figure 2b), which may allow for different removal pathways during the coagulation–flocculation process [27]. The observed particle size is comparable to that reported for other biopolymers, where particles smaller than 20 µm have been described as having a greater adsorption potential, while particles close to 100 µm are mainly associated with polymer bridging mechanisms, facilitating the formation of larger aggregates that settle more rapidly an expected behavior in natural coagulants based on polysaccharides [47,48].

Likewise, particles in the 50–100 µm range tend to show adequate dispersion in the aqueous medium and favor physical processes such as interception and sedimentation [53]. In contrast, smaller particles around 5 µm, due to their larger surface area, can enhance adsorption processes [54].

The bimodal particle size of CNS could be due to the extraction conditions of the biopolymer, which, when interacting with water, tends to agglomerate, forming larger particles. Furthermore, this behavior may be related to the degradation of proteins and carbohydrates, which, when dissolved in water, release particles of different sizes [34,55,56]. Meanwhile, CMN exhibited a particle size between 50 and 100 µm, similar to that reported for *Opuntia ficus-indica*, with an average size of 166 µm [46,47,57,58]. However, the efficiency of the process can be influenced by an appropriate particle size, since excessively large particles can settle quickly and reduce the stability of the dispersion, while smaller particle sizes are associated with better performance of the coagulation process [59,60].

#### 3.2.3. Zeta Potential (ζ) and Point of Zero Charge (PZC) of the Biocoagulants

**Figure 3:**
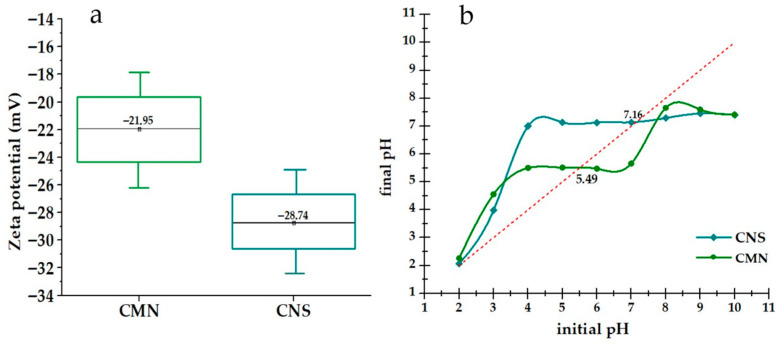
(**a**) Zeta potential (ζ) of CNS and CMN; (**b**) determination of the point of zero charge (PZC) of CNS and CMN as a function of initial and final pH. The dashed line represents the condition initial pH = final pH, where the PZC is identified.

As shown in Figure 3a, the ζ potential values indicate that both biocoagulants exhibit an anionic surface charge. CNS showed a more negative value (−28.74 mV) compared to CMN (−21.95 mV). This suggests that CNS may present higher colloidal stability and longer polysaccharide chains, which could favor particle bridging phenomena, as reported in previous studies [61,62,63,64]. On the other hand, the less negative potential of CMN indicates reduced electrostatic repulsion, which may enhance surface interactions and contribute to adsorption-related processes [48,65,66]. About the PZC, Figure 3b shows values of 7.16 for CNS and 5.49 for CMN. Both materials acquire a negative charge when the pH exceeds their PZC. Under these conditions, the negative surface charge may promote interactions with cationic species and contribute to floc formation through mechanisms such as polymer bridging and adsorption, involving carboxyl (-COOH) and hydroxyl (-OH) functional groups, which may play a role in metal removal [5,67,68,69,70].

### 3.3. Analysis of Experimental Data

In Table S1 (Supplementary Materials), it was observed that treatment with pure CSA achieved a turbidity reduction of 46.20 NTU. However, higher doses caused acidification of the leachate and an increase in electrical conductivity (EC). In contrast, treatments combining CSA with CNS and CMN achieved comparable turbidity reductions of up to 48 NTU using lower doses of CSA, while at the same time maintaining the pH and EC of the leachate.

Regarding EC, its behavior was influenced by the CSA dose, with higher doses increasing EC above 13 mS/cm, which suggests the incorporation of Al^3+^ and SO_4_^2−^ into the leachate due to the dissolution of aluminum sulfate [1,23,45]. In contrast, the biocoagulants did not cause an increase in EC.

The initial pH of the leachate decreased from 8.01 to 5.28 with high doses of CSA. This decrease could be associated with the typical behavior of leachates with reduced buffer capacity in the face of Al^3+^ hydrolysis, a process that releases protons and contributes to the acidification of the medium [14]. Meanwhile, incorporating CNS and CMN attenuated this effect, maintaining the pH close to neutrality, which is consistent with their capacity to partially buffer the acidification induced by metal salts.

On the other hand, leachates usually present a complex composition dominated by organic acids and limited alkalinity, which translates into a low buffering capacity against the addition of acidic species [5,71].

Likewise, rapid pH drops in leachates have been observed both from the addition of acidic coagulants and from the production of volatile organic acids, especially in young leachates [72]. This represents an advantage in leachate treatment, since these usually require additional pH corrections when metal salt coagulants are used [19].

### 3.4. Model Adequacy Verification

The models obtained for turbidity, pH, and EC presented R^2^ values higher than 0.91 (Table 4), indicating good agreement between the experimental and modeled data. The differences between the adjusted and predicted R^2^ values were below the recommended limit of 0.20 (pH: 0.135; EC: 0.059; turbidity: 0.184), supporting the predictive reliability of the models [64].

In addition, the adequate precision values exceeded the minimum acceptable value of 4.0, reaching 14.66 for pH, 25.27 for EC, and 13.93 for turbidity, which reflects a satisfactory signal-to-noise ratio and a clear identification of the effects of the studied factors [41,43,73,74].

However, a statistically significant lack of fit was observed for turbidity and EC, indicating differences between the model predictions and some experimental values. This behavior can be attributed to the sensitivity of the Box–Behnken design to detect small deviations [41,74]. Furthermore, this lack of fit may be associated with the high complexity and variability inherent to landfill leachate, whose composition varies over time and is influenced by the interaction among its different components [5,75]. Under these conditions, the ability of quadratic models to accurately describe the behavior of the responses may be limited, since they assume smooth and continuous relationships, whereas in practice the interactions between leachate components and small changes in dosages or operating conditions during the coagulation and flocculation process may lead to pronounced curvature, inflection points, or saturation effects that are not always adequately captured by a quadratic model [1,38,43].

**Figure 4:**
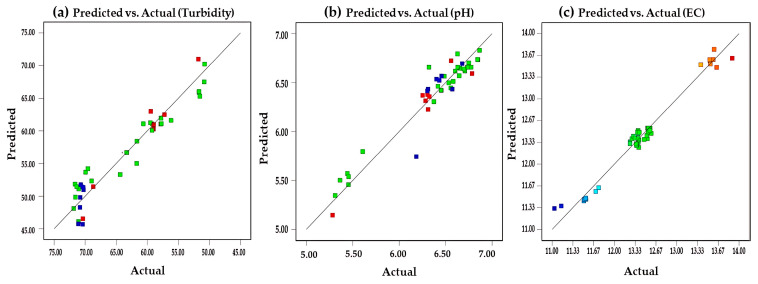
Predicted versus experimental values for turbidity, pH, and electrical conductivity, with color-coded experimental values.

On the other hand, the models were employed as empirical tools to identify trends and approximate optimal regions within the evaluated experimental range. The validity of the optimal conditions was confirmed through experimental validation, which yielded results within the range of values observed during the experimentation. Furthermore, the model performance was further supported by the close agreement between predicted and experimental values shown in Figure 4, confirming the adequacy of the models within the studied experimental domain [76].

### 3.5. Response Surface Analysis for Turbidity

**Figure 5:**
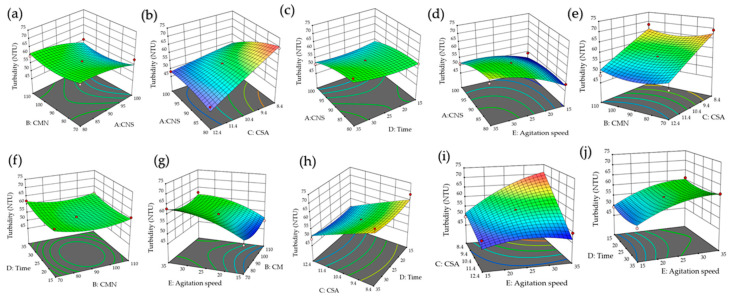
Response surface plots showing the combined effects of CNS, CMN, CSA, mixing time, and agitation speed on leachate turbidity: (**a**) CNS vs. mixing time; (**b**) CNS vs. agitation speed; (**c**) CMN vs. mixing time; (**d**) CMN vs. agitation speed; (**e**) CSA vs. mixing time; (**f**) CSA vs. agitation speed; (**g**) CNS vs. CMN; (**h**) CNS vs. CSA; (**i**) CMN vs. CSA. (**j**) mixing time–agitation speed.

The response surface plots shown in Figure 5 indicate a complementary interaction between aluminum sulfate (CSA) and the biopolymers, where CNS and CMN contribute to turbidity reduction through adsorption and polymer bridging mechanisms (Figure 5a,b,e). In addition, moderate mixing times and agitation speeds favor floc stability, whereas excessive agitation or prolonged mixing increases residual turbidity due to floc breakage caused by shear forces (Figure 5c,d,g,j) [74].

Furthermore, increasing CMN dosage enhances turbidity removal across different mixing conditions, highlighting the combined influence of coagulant dosage and hydrodynamic conditions on clarification performance (Figure 5f,h,i).

### 3.6. Optimization

**Figure 6:**
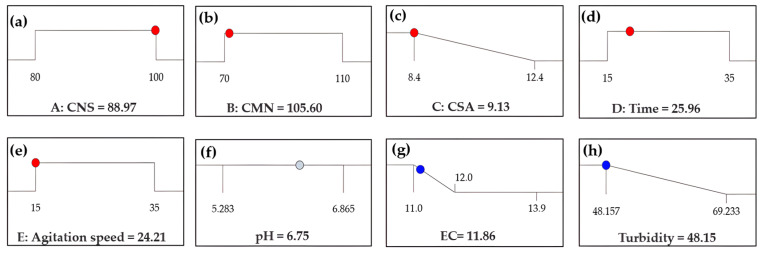
Optimal operating conditions and predicted responses obtained by the Box–Behnken design: (**a**–**e**) CNS, CMN, and CSA dosages, mixing time, and agitation speed; (**f**–**h**) predicted pH, electrical conductivity, and turbidity.

For the optimization, the doses of CNS, CMN, and CSA, as well as the mixing time and agitation speed, were considered. The response variable was turbidity reduction, with the constraints of maintaining the pH close to neutrality and the electrical conductivity equal to or lower than the initial value, in order to avoid alterations in the leachate. The optimization reduced turbidity to 48.15 NTU, using doses of 88.97 mg/L of CNS, 105.60 mg/L of CMN, and 9.13 mg/L of CSA, with a mixing time of 25.96 min and an agitation speed of 24.21 rpm. Under these conditions, the pH was maintained at 6.75 and the electrical conductivity at 11.86 mS/cm, as shown in Figure 6. The turbidity value achieved a decline within the experimental range of 48 to 69 NTU (see Table S1, Supplementary Materials).

### 3.7. Experimental Validation of the Optimal Treatment

To validate the optimal conditions determined using the Box–Behnken design, coagulation–flocculation tests were carried out using new leachate samples characterized in Table 5. The experiments were conducted under the optimal conditions predicted by the model. Under these conditions, turbidity was reduced to 49.02 NTU, achieving a removal efficiency of 70%, as shown in Table 5, a value comparable to that reported for natural biocoagulants (50–80%) [5,21,23,44,74].

The turbidity obtained during the experimental validation was lower than that recorded during the optimization stage (48.15 NTU), as shown in Figure 6. This difference can be attributed to variations in the physicochemical characteristics of the leachate used in the validation, since, although the samples were collected at the same location, they were obtained at different times (Table 5). Nevertheless, the experimental result remained within the turbidity range observed during the experimental runs (see Table S1, Supplementary Materials).

This performance is associated with the anionic nature of both biocoagulants, as evidenced by negative zeta potential values of −28.74 mV for CNS and −21.95 mV for CMN; these results suggest that charge neutralization is not the dominant mechanism, and that contaminant removal may be primarily associated with adsorption and polymer bridging phenomena, rather than complete electrostatic neutralization [1,40,77]. This interpretation is supported by the PZC values of CNS (7.16) and CMN (5.49), which indicate that both biocoagulants maintain a negative surface charge under near-neutral pH conditions [68,69]. Furthermore, the particle size distribution of CNS, characterized by differentiated fractions (<10 µm and 100 µm), together with the more homogeneous particle size of CMN, may favor adsorption processes and floc growth, contributing to turbidity reduction. These effects are consistent with previous studies, which have shown that particle size distribution and surface charge heterogeneity influence floc formation and aggregation behavior during coagulation–flocculation processes [9,26,78,79].

Regarding organic matter removal, TOC was reduced by 65.3%, while COD and BOD_5_ showed more limited reductions of 14.7% and 24.7%, respectively. This behavior is consistent with the role of coagulation–flocculation as a pretreatment process, which preferentially removes colloidal and macromolecular organic matter rather than dissolved low-molecular-weight compounds, while part of the organic fraction remains dissolved in the leachate [80,81].

On the other hand, phosphate removal reached 47.7%, suggesting that part of the phosphate binds to the flocs formed during treatment, while the remainder stays in the leachate. In contrast, ammoniacal nitrogen was reduced to only 12.5%, which could be attributed to the fact that the NH_4_^+^ ion remains mostly dissolved and exhibits limited interaction with anionic biocoagulants [75,82,83]. These results confirm that the mixture used does not completely remove ammoniacal nitrogen and instead serves as a conditioning step prior to biological or advanced nitrogen removal processes.

Metal removal efficiencies varied, reaching up to 67.7% for Cu, 55.9% for As, and 40% for Cd. This behavior is attributed to adsorption and chelation mechanisms associated with the carboxyl and hydroxyl functional groups present in the biocoagulants [11,26,84,85]. In contrast, Cr, Fe, Pb, Zn, Al, and Ni showed lower removal efficiencies (19–39%), probably due to competition among cations present in the leachate [44,86].

Finally, surfactant removal reached 19.465%, indicating that most of the surfactants remained dissolved [87]. The combined use of CNS–CMN biocoagulants with aluminum sulfate acts mainly as a physicochemical pretreatment, selectively removing turbidity, colloidal organic matter, and metals, while most dissolved contaminants remain in the leachate, supporting its role as a preliminary step prior to biological or advanced treatments.

## 4. Conclusions

This study used a combination of the biocoagulants CNS and CMN with aluminum sulfate (CSA) for the treatment of sanitary landfill leachate through a coagulation–flocculation process. The physicochemical characterization of both biocoagulants showed that they present carboxyl and hydroxyl functional groups and have an anionic nature. The optimal conditions determined by the model were 88.97 mg/L of CNS, 105.60 mg/L of CMN, and 9.13 mg/L of CSA. Furthermore, operating intervals for mixing time were established between 25 and 30 min, with an agitation speed of 20 to 25 rpm. These conditions allowed for a predicted turbidity reduction to 48.15 NTU; during validation, the turbidity was reduced to 49.02 NTU, achieving removal efficiencies within the 70% range. Furthermore, TOC reductions of 65% were achieved, and heavy metals such as arsenic (As) and cadmium (Cd) reached removal efficiencies of 56% and 40%, respectively. Phosphate removal was moderate, while the removal of BOD_5_, surfactants, and ammonia nitrogen was limited. These findings indicate that biocoagulants constitute an efficient pretreatment for leachates and could serve as sustainable aids, reducing dependence on synthetic coagulants.

## Figures and Tables

**Table 1:** Analytical methods used for leachate characterization.

Parameters	Method	Unit	Reference
pH	Selective electrode	-	Standard Methods 4500—H + B [32]
Electrical Conductivity (EC)	Selective electrode	mS/cm	Standard Methods 2510 B [32]
Turbidity	Nephelometry	NTU	Standard Methods 2130 B [32]
Biochemical Oxygen Demand (BOD_5_)	Respirometric, manometric oxytope method	mg/L	Standard Methods 5210D [32]
Chemical Oxygen Demand (COD)	Closed reflux, colorimetric method	mg/L	Standard Methods 5220B [32]
Metals (Fe, Cu, Cd, Zn, Pb, Cr, As, Ni, Al)	Optical emission spectrometry	mg/L	ICP-OES 9820, Standard Methods 3120 B [32]
Total Organic Carbon (TOC)	Catalytic combustion	mg/L	Standard Methods 5310 B [32]
Phosphates	Ascorbic acid method	mg/L	Standard Methods 4500-P E [32]
Ammonia Nitrogen (NH_3_-N)	Nessler method	mg/L	ASTM Standards, D 1426 [33]
Surfactants	Methylene blue method	mg/L	Standard Methods 5540 C [32]

**Table 2:** Variables and levels of the Box–Behnken design.

Variable	Symbol	−1	0	1
CNS (mg/L)	A	80	90	100
CMN (mg/L)	B	70	90	110
CSA (mg/L)	C	8.4	10.4	12.4
Mixing time (min)	D	15	25	35
Agitation speed (rpm)	E	15	25	35

**Table 3:** Initial quality parameters of the untreated leachate.

Parameters	$\bar{x}$	±S	Limit mg/L
PH	8.01	0.00	-
EC (mS/cm)	12.6	0.00	-
Turbidity (NTU)	164.8	0.05	-
NH_3_-N (mg/L)	640.0	0.05	5 [44]
Phosphates (mg/L)	0.23	0.00	-
Surfactants (mg/L)	0.31	0.00	-
COD (mg/L)	817	0.00	400 [44]
BOD_5_ (mg/L)	563	0.58	20 [44]
BOD_5_/COD	0.69	-	-
TOC (mg/L)	252.3	0.06	-
As (mg/L)	0.75	0.01	0.050 [44]
Cd (mg/L)	0.48	0.01	0.010 [44]
Cu (mg/L)	0.57	0.01	0.020 [44]
Cr (mg/L)	0.38	0.00	0.010 [44]
Fe (mg/L)	0.31	0.00	0.005 [44]
Pb (mg/L)	0.38	0.00	0.010 [44]
Zn (mg/L)	0.20	0.01	2 [44]
Al (mg/L)	0.35	0.00	-
Ni (mg/L)	0.05	0.00	-

$\bar{x}$ is mean; S is standard deviation.

**Table 4:** Coefficients of the quadratic regression models for turbidity, pH, and EC.

Coefficient	Turbidity	*p*-Value	pH	*p*-Value	EC	*p*-Value
Intercept	56.687	-	6.656	-	12.551	-
A	−3.507	<0.0001	0.001	0.9764	0.010	0.7861
B	−0.467	0.4936	−0.027	0.5251	0.025	0.4974
C	−7.557	<0.0001	−0.567	<0.0001	1.061	<0.0001
D	0.460	0.4999	−0.077	0.0817	0.001	0.9770
E	5.102	<0.0001	0.157	0.0011	−0.091	0.0173
AB	−0.384	0.7778	−0.034	0.6900	0.053	0.4690
AC	4.508	0.0026	0.033	0.7014	0.000	1.0000
AD	−1.134	0.4072	−0.005	0.9513	0.051	0.4831
AE	−2.250	0.1067	−0.001	0.9954	−0.003	0.9723
BC	0.274	0.8405	−0.014	0.8729	0.011	0.8806
BD	0.025	0.9853	0.048	0.5744	−0.005	0.9447
BE	−0.250	0.8540	−0.106	0.2258	−0.038	0.5961
CD	0.175	0.8973	−0.222	0.0151	−0.140	0.0611
CE	−4.683	0.0018	0.068	0.4322	0.040	0.5803
DE	−0.125	0.9267	0.027	0.7580	−0.012	0.8715
A2	−1.787	0.0608	−0.056	0.3364	−0.074	0.1403
B2	2.756	0.0056	−0.094	0.1136	−0.097	0.0565
C2	−0.237	0.7970	−0.497	<0.0001	0.054	0.2731
D2	1.603	0.0905	−0.149	0.0160	−0.045	0.3579
E2	−2.786	0.0052	−0.022	0.7105	−0.093	0.0672
Lack of fit		<0.0001		0.5674		<0.0001
R^2^	-	0.9165	-	0.9194	-	0.9732
Adjusted R^2^	-	0.8496	-	0.8549	-	0.9517
Adequate Precision	-	13.9333	-	14.6613	-	25.2733
predicted R^2^	-	0.6658		0.7195	-	0.8929

A–E correspond to CNS, CMN, CSA, mixing time (min), and agitation speed (rpm), respectively.

**Table 5:** Removal performance of leachate parameters under optimal treatment conditions using CNS, CMN, and CSA.

Parameters	Initial			Treatment (CNS, CMN, CSA)			*t*-Test Significance	%R
	$\bar{x}$	±S	CV	$\bar{x}$	±S	CV		
PH	8.02	0.00	0.00	6.47	0.03	0.45	**	--
EC (mS/cm)	12.61	0.00	0.00	11.45	0.00	0.01	**	9.2
Turbidity (NTU)	163.52	0.01	0.00	49.02	0.02	0.04	**	70.0
NH_3_-N (mg/L)	640.03	0.05	0.01	560.25	0.50	0.09	**	12.5
Phosphates (mg/L)	0.23	0.00	0.25	0.12	0.00	0.83	**	47.7
Surfactants (mg/L)	0.31	0.00	0.19	0.25	0.00	0.40	**	19.5
COD (mg/L)	816.99	0.01	0.00	697.25	0.50	0.07	**	14.7
BOD_5_ (mg/L)	563.33	0.58	0.10	424.00	0.00	0.00	**	24.7
BOD_5_/COD	0.69	-	-	0.61	-	-	-	-
TOC (mg/L)	252.20	0.17	0.07	87.60	0.00	0.00	**	65.3
As (mg/L)	0.75	0.01	0.77	0.33	0.00	0.02	**	55.9
Cd (mg/L)	0.48	0.00	0.12	0.29	0.00	0.00	**	40.0
Cu (mg/L)	0.57	0.00	0.10	0.19	0.00	0.83	**	67.7
Cr (mg/L)	0.38	0.00	0.00	0.23	0.00	0.00	**	39.2
Fe (mg/L)	0.31	0.00	0.19	0.20	0.00	0.00	**	36.6
Pb (mg/L)	0.38	0.00	0.00	0.30	0.00	0.19	**	20.8
Zn (mg/L)	0.20	0.00	0.36	0.16	0.00	0.37	**	19.6
Al (mg/L)	0.35	0.00	0.17	0.28	0.00	0.00	**	19.8
Ni (mg/L)	0.05	0.00	0.00	0.04	0.00	0.17	**	35.2

$\bar{x}$ mean; S: standard deviation; CV: coefficient of variation; %R: removal efficiency. ** indicates statistically significant differences according to the paired Student’s *t*-test (*p* < 0.05). Values reported as 0.00 in ±S indicate variations below the resolution or rounding of the measurement method.

## Data Availability

The original contributions presented in this study are included in the article. Further inquiries can be directed to the corresponding author.

## Supplementary Materials

The following supporting information can be downloaded at: https://www.mdpi.com/article/10.3390/polym18040474/s1; Table S1: Experimental matrix for turbidity, pH, and EC responses.
