# Supplementary material for: Application of Nostoc sphaericum and Opuntia ficus-indica Mucilage in the Coagulation–Flocculation Process of Sanitary Landfill Leachate: An Optimization Study
Source: Polymers (Basel). 2026 Feb 13;18(4):474. doi: 10.3390/polym18040474 (PMC12944250; doi:10.3390/polym18040474)
Supplement: Supplementary file 1 [file polymers-18-00474-s001.zip › polymers-4119821-supplementary.pdf]

Table S1. Experimental matrix for turbidity, pH, and EC responses

Run	CNS dose (mg/L)	CMN dose (mg/L)	CSA dose (mg/L)	Mixing time (min)	Agitation speed (rpm)	Experimental values			Predicted values		
						pH	EC (mS/cm)	Turbidity (NTU)	pH	EC (mS/cm)	Turbidity (NTU)
**Inicial**						**8.0143**	**12.707**	**164.775**			
CSA	0	0	12.40	35	15	5.10	13.45	46.20			
	0	0	10.40	35	15	5.70	12.98	48.70			
	0	0	8.40	35	15	5.94	12.71	49.06			
**1**	100	90	10.40	25	15	6.45	12.40	48.90	6.42	12.49	45.76
**2**	80	90	10.40	25	15	6.45	12.38	49.17	6.42	12.46	48.27
**3**	90	90	12.40	25	15	5.31	13.89	49.60	5.35	13.62	45.69
**4**	90	90	8.40	25	15	6.60	11.70	49.47	6.62	11.58	51.44
**5**	90	90	10.40	35	15	6.31	12.38	49.73	6.23	12.52	50.99
**6**	90	90	10.40	15	15	6.31	12.37	49.20	6.43	12.49	49.82
**7**	90	110	10.40	25	15	6.42	12.57	49.70	6.46	12.52	51.34
**8**	90	70	10.40	25	15	6.37	12.38	49.30	6.31	12.39	51.77
**9**	90	90	10.40	25	25	6.74	12.54	56.69	6.66	12.55	56.69
**10**	90	90	10.40	25	25	6.74	12.53	56.69	6.66	12.55	56.69
**11**	90	90	10.40	25	25	6.63	12.55	56.69	6.66	12.55	56.69
**12**	90	90	10.40	25	25	6.73	12.56	56.69	6.66	12.55	56.69
**13**	90	90	10.40	25	25	6.77	12.54	56.69	6.66	12.55	56.69
**14**	90	90	10.40	25	25	6.32	12.57	56.69	6.66	12.55	56.69
**15**	90	110	10.40	35	25	6.33	12.30	60.80	6.36	12.43	61.07
**16**	90	70	10.40	35	25	6.28	12.28	62.20	6.31	12.39	61.95
**17**	90	110	10.40	15	25	6.30	12.52	60.77	6.41	12.44	60.09
**18**	90	70	10.40	15	25	6.46	12.48	62.27	6.57	12.38	61.08
**19**	100	90	10.40	35	25	6.25	12.54	51.03	6.37	12.49	52.32
**20**	80	90	10.40	35	25	6.30	12.40	63.83	6.38	12.37	61.60
**21**	100	90	10.40	15	25	6.40	12.53	50.07	6.53	12.39	53.67
**22**	80	90	10.40	15	25	6.43	12.59	58.33	6.52	12.47	58.42
**23**	90	110	12.40	25	25	5.45	13.59	48.63	5.46	13.60	51.46
**24**	90	70	12.40	25	25	5.46	13.54	48.37	5.54	13.53	51.84
**25**	90	110	8.40	25	25	6.71	11.51	68.33	6.62	11.46	66.02
**26**	90	70	8.40	25	25	6.65	11.51	69.17	6.65	11.43	67.50
**27**	100	90	12.40	25	25	5.44	13.53	48.16	5.57	13.60	48.11

Run	CNS dose (mg/L)	CMN dose (mg/L)	CSA dose (mg/L)	Mixing time (min)	Agitation speed (rpm)	Experimental values			Predicted values		
						pH	EC (mS/cm)	Turbidity (NTU)	pH	EC (mS/cm)	Turbidity (NTU)
**28**	80	90	12.40	25	25	5.36	13.53	48.93	5.50	13.58	46.11
**29**	100	90	8.40	25	25	6.70	11.54	50.43	6.64	11.48	54.21
**30**	80	90	8.40	25	25	6.75	11.54	69.23	6.70	11.46	70.23
**31**	90	90	12.40	35	25	5.28	13.64	48.97	5.14	13.48	51.13
**32**	90	90	8.40	35	25	6.56	11.75	68.30	6.72	11.64	65.90
**33**	90	90	12.40	15	25	6.18	13.60	48.43	5.74	13.76	49.86
**34**	90	90	8.40	15	25	6.57	11.15	68.47	6.43	11.36	65.33
**35**	100	110	10.40	25	25	6.56	12.38	55.63	6.45	12.47	53.30
**36**	80	110	10.40	25	25	6.58	12.25	59.37	6.51	12.34	61.08
**37**	100	70	10.40	25	25	6.65	12.25	58.30	6.57	12.31	55.00
**38**	80	70	10.40	25	25	6.53	12.33	60.50	6.50	12.40	61.25
**39**	100	90	10.40	25	35	6.84	12.36	51.30	6.74	12.30	51.46
**40**	80	90	10.40	25	35	6.85	12.35	60.57	6.73	12.29	62.97
**41**	90	90	12.40	25	35	5.61	13.39	49.63	5.80	13.52	46.53
**42**	90	90	8.40	25	35	6.63	11.04	68.23	6.79	11.32	71.01
**43**	90	90	10.40	35	35	6.78	12.35	61.03	6.59	12.31	60.94
**44**	90	90	10.40	15	35	6.68	12.38	61.00	6.69	12.33	60.27
**45**	90	110	10.40	25	35	6.49	12.39	62.17	6.56	12.26	61.04
**46**	90	70	10.40	25	35	6.87	12.35	62.77	6.83	12.28	62.48
